# H3K27me3 demethylases regulate in vitro chondrogenesis and chondrocyte activity in osteoarthritis

**DOI:** 10.1186/s13075-016-1053-7

**Published:** 2016-07-07

**Authors:** Clarence Yapp, Andrew J. Carr, Andrew Price, Udo Oppermann, Sarah J. B. Snelling

**Affiliations:** Nuffield Department of Orthopaedics, Rheumatology and Musculoskeletal Sciences, Botnar Research Centre, University of Oxford, Nuffield Orthopaedic Centre, Windmill Road, Headington, OX3 7LD Oxford, UK; Structural Genomics Consortium, University of Oxford, Oxford, UK; Oxford Stem Cell Institute, Oxford, UK

**Keywords:** Epigenetics, TGF-β, Osteoarthritis, JMJD3, Histone demethylase

## Abstract

**Background:**

Epigenetic changes (i.e., chromatin modifications) occur during chondrogenesis and in osteoarthritis (OA). We investigated the effect of H3K27me3 demethylase inhibition on chondrogenesis and assessed its utility in cartilage tissue engineering and in understanding cartilage destruction in OA.

**Methods:**

We used a high-content screen to assess the effect of epigenetic modifying compounds on collagen output during chondrogenesis of monolayer human mesenchymal stem cells (MSCs). The impact of GSK-J4 on gene expression, glycosaminoglycan output and collagen formation during differentiation of MSCs into cartilage discs was investigated. Expression of lysine (K)-specific demethylase 6A (*UTX*) and Jumonji domain-containing 3 (*JMJD3*), the HEK27Me3 demethylases targeted by GSK-J4, was measured in damaged and undamaged cartilage from patients with OA. The impact of GSK-J4 on ex vivo cartilage destruction and expression of OA-related genes in human articular chondrocytes (HACs) was assessed. H3K27Me3 demethylase regulation of transforming growth factor (TGF)-β-induced gene expression was measured in MSCs and HACs.

**Results:**

Treatment of chondrogenic MSCs with the H3K27me3 demethylase inhibitor GSK-J4, which targets JMJD3 and UTX, inhibited collagen output; expression of chondrogenic genes, including *SOX9* and *COL2A1*; and disrupted glycosaminoglycan and collagen synthesis. *JMJD3* but not *UTX* expression was increased during chondrogenesis and in damaged OA cartilage, suggesting a predominant role of JMJD3 in chondrogenesis and OA. GSK-J4 prevented ex vivo cartilage destruction and expression of the OA-related genes *MMP13* and *PTGS2.* TGF-β is a key regulator of chondrogenesis and articular cartilage homeostasis, and TGF-β-induced gene expression was inhibited by GSK-J4 treatment of both chondrogenic MSCs and HACs.

**Conclusions:**

Overall, we show that H3K27me3 demethylases modulate chondrogenesis and that enhancing this activity may improve production of tissue-engineered cartilage. In contrast, targeted inhibition of H3K27me3 demethylases could provide a novel approach in OA therapeutics.

**Electronic supplementary material:**

The online version of this article (doi:10.1186/s13075-016-1053-7) contains supplementary material, which is available to authorized users.

## Background

Osteoarthritis (OA) is the most common of the arthritides and is characterized by loss of articular cartilage. Treatments for OA are limited to pain relief, physiotherapy and joint replacement surgery for end-stage disease. To successfully treat cartilage lesions and OA, it is paramount to improve production of tissue-engineered cartilage and to identify disease-modifying therapeutics that can prevent or limit cartilage degradation.

Gene expression and cellular phenotype changes associated with OA and chondrogenesis are increasingly being attributed to altered activity of epigenetic modifying enzymes and consequent epigenetic regulation of target genes [1, 2]. Commonly, epigenetic modifications occur in gene promoters and include regulation of chromatin structure through cytosine methylation of DNA or through modifications such as acetylation and methylation of lysine residues in histone tails. Pharmacological intervention points in oncology have been identified by targeting of “readers, writers and erasers” of an “epigenetic code” [3], an avenue with largely unexplored potential in inflammatory and degenerative diseases.

Correct regulation of transforming growth factor (TGF)-β signalling is essential for cartilage development [4]. Dysregulation of TGF-β pathway components leads to impaired skeletogenesis, increased chondrocyte hypertrophy and an OA-like phenotype in murine models [5, 6]. Furthermore a disrupted TGF-β signalling response in both murine and human OA has been reported [7]. Epigenetic regulation of the TGF-β pathway in fibrosis has been reported, and TGF-β itself regulates expression of epigenetic modifying enzymes [8].

We reasoned that specific targeting of epigenetic modifying enzymes could provide valuable insight into the role of epigenetics in chondrogenesis and OA and help identify exploitable targets for therapeutic development. In this work, we identified and investigated the H3K27me3 demethylases as novel targets in cartilage tissue engineering and OA pathogenesis.

## Methods

### Cell culture and tissue preparation

Primary human bone marrow-derived mesenchymal stem cells (MSCs) were purchased from Lonza (Walkersville, MD, USA) and cultured in MSC expansion media consisting of MesenPRO RS basal media with MesenPRO RS supplement, 2 mM l-glutamine, 100 IU ml^−1^ penicillin and 100 mg ml^−1^ streptomycin (Life Technologies, Carlsbad, CA, USA). MSCs were used for all experiments before passage 7.

Human articular cartilage for gene expression analysis was from damaged and undamaged regions of the medial tibial plateau of individuals undergoing unicompartmental knee replacement for anteromedial OA (*n* = 5 patients). For cell culture, cartilage from the medial tibial plateau of patients undergoing unicompartmental knee replacement for OA was dissected and digested overnight with collagenase. HACs were cultured in basal medium composed of high-glucose DMEM (Lonza) containing 2 mM glutamine and 100 IU ml^−1^ penicillin and 100 mg ml^−1^ streptomycin (Life Technologies).

Ethical approval was granted (09/H0606/11) by the local research ethics committee (Oxford Research Ethics Committee B) for all work on human articular cartilage and chondrocytes, and informed consent was obtained from all patients.

### Chondrogenic differentiation

For monolayer chondrogenesis, MSCs in expansion media were seeded at 20,000 cells/well in a 96-well plate and left to adhere overnight. Expansion media were replaced with high-glucose DMEM (Lonza), chondrogenic differentiation media containing 100 μg/ml sodium pyruvate (Lonza), 10 ng/ml TGF-β3 (R&D Systems, Minneapolis, MN, USA), 100 nM dexamethasone, 1× ITS+ premix (BD Biosciences, San Jose, CA, USA), 40 μg/ml proline, and 25 μg/ml ascorbate 2-phosphate (Sigma-Aldrich, St. Louis, MO, USA). Media were refreshed every 2–3 days.

For cartilage disc generation, MSCs were resuspended in chondrogenic medium and 100 μl of MSCs at 5 × 10^6^ cells/ml were pipetted onto 6.5-mm, 0.8-μm pore polycarbonate Transwell filters (Corning Costar, Corning, NY, USA) in a 24-well plate, centrifuged (200 × *g*, 5 min) before addition of 500 μl of chondrogenic medium to the lower well. Media were refreshed every 2–3 days.

### Compound screen

Using a focused library of 31 small-molecule inhibitors against various readers, writers and erasers of chromatin modifications, we assessed chondrogenic differentiation of MSCs. Chondrogenic monolayer MSCs were incubated for 7 days in the presence or absence of epigenetic modifying compounds, where compounds and media were refreshed every 2–3 days. Cell viability was assessed using the alamarBlue assay (Life Technologies) before fixing cells in 4 % formaldehyde and staining with 10 μg/ml Nile Red, which binds non-specifically to collagens and lipids and allows visualization of chondrogenic nodules [9]. Nile Red staining intensity was assessed using excitation at 485 nm in the BD Pathway (BD Biosciences). Data are presented as Nile Red incorporation relative to cell viability (Additional file 1: Figure S1).

### Cytokine and GSK-J4 treatments

Primary HACs were seeded at 6000 cells/well in a 96-well plate and allowed to adhere overnight. HACs were treated with GSK-J4 (10 μM), interleukin (IL)-1β (5 ng/ml), IL-6 (10 ng/ml), oncostatin M (OSM) (10 ng/ml), TGF-β (4 ng/ml) or TGF-β and GSK-J4. Dimethyl sulphoxide (DMSO) alone was added to all wells not containing GSK-J4. MSCs were seeded at 20,000 cells/well of a 96-well plate and allowed to adhere overnight in MSC expansion medium before 1- and 24-h treatment in basal, chondrogenic (plus DMSO carrier) and chondrogenic medium plus GSK-J4. Treated HACs and MSCs were washed twice in PBS, and cells were harvested in cells to complementary DNA (cDNA) lysis buffer (Ambion; Thermo Fisher Scientific, Austin, TX, USA) prior to cDNA synthesis. GSK-J5 (10 μM), the less active enantiomer of GSK-J4, was used as an additional comparator for experiments (Additional file 1: Figure S1 and Additional file 2: Figure S3).

### Small interfering RNA

HACs and MSCs were transfected with 5 nM (final concentration) of small interfering (siRNA) against Jumonji domain-containing 3 (*JMJD3*) and lysine (K)-specific demethylase 6A (*UTX*) (QIAGEN, Hilden, Germany) or AllStars non-targeting negative control (QIAGEN) using DharmaFECT reagent (GE Healthcare, Little Chalfont, UK) according to the manufacturer’s instructions. MSCs were pre-treated with siRNA for 48 h prior to induction of chondrogenesis. HACs were transfected with siRNA 72 h prior to harvest for gene expression analysis.

### Gene expression analysis

Following cytokine treatment of monolayer HACs and MSCs, cell-to-cDNA lysates were treated with DNase and synthesized with cDNA using Moloney murine leukaemia virus (MMLV) reverse transcriptase (Life Technologies) according to the manufacturer’s instructions. MSC cartilage discs were harvested in TRIzol reagent (Life Technologies) and ground using disposable plastic pestles and Molecular Grinding Resin (G-Biosciences, St. Louis, MO, USA). Human articular cartilage was snap frozen, crushed and ground in liquid nitrogen using a pestle and mortar before RNA extraction using TRIzol reagent (Life Technologies). Total RNA was converted to cDNA using MMLV reverse transcriptase (Life Technologies) according to the manufacturer’s instructions.

Expression of genes of interest was measured by quantitative reverse transcription polymerase chain reaction (RT-qPCR) on an Applied Biosystems ViiA 7 system using SYBR Green (Life Technologies). Relative quantification is expressed as the comparative cycle threshold (2^−ΔCt^), where ΔC_t_ is C_t_(gene of interest) − C_t_(reference gene *TBP* or *GAPDH*). Samples where the reference gene C_t_ was greater than ±1.5 C_t_ from the median were excluded from further analyses.

### Immunocytochemistry

MSCs and HACs were seeded in chamber slides and allowed to adhere overnight. MSCs were treated with control medium (DMSO alone) or chondrogenic medium with or without GSK-J4 (10 μM). HACs were treated with control medium (DMSO alone) or TGF-β with or without GSK-J4. After 24-h (MSCs) and 6-h treatment (HAC), cells were fixed in 4 % formaldehyde and stained overnight at 4 °C with anti-H3K27Me3 (5 μg/ml, catalog number ab6002; Abcam, Cambridge, UK) before 1-h incubation at room temperature with rabbit antimouse immunoglobulin G. Nuclei were visualized using 4′,6-diamidino-2-phenylindole stain, and the cell cytoskeleton was visualized with Texas Red-conjugated phalloidin (Life Technologies). Images were obtained using a Zeiss inverted microscope using AxioVision software (Carl Zeiss Microscopy, Thornwood, NY, USA).

### Dimethylmethylene blue assay for glycosaminoglycan content

Primary human articular cartilage explants were incubated with 5 ng/ml IL-1 and 10 ng/ml OSM in the presence or absence of GSK-J4 (10 μM) for 7 days, and media were collected and harvested every 2–3 days. Cartilage explants at day 7 and MSC cartilage discs at 14 days of differentiation were digested with papain overnight. Aliquots of media and digested cartilage were mixed with 1,9-dimethylmethylene blue and immediately read at 630 nm (SpectraMax Plus 384; Molecular Devices, Sunnyvale, CA, USA).

### Second harmonic generation

Z-stack images were acquired on a Zeiss LSM 710 NLO confocal scanning microscope (Carl Zeiss Microscopy) coupled to a Chameleon Vision II multiphoton laser (Coherent, Santa Clara, CA, USA) tuned to 900 nm. Second harmonic generation (SHG) was confirmed at precisely 450 nm using a spectral detector and detected between 370 and 480 nm using non-descanned detectors through a × 25, 0.8 numerical aperture glycerol immersion objective. The stack thickness was 80–100 μm with a step size of 1.39 μm.

### Statistical analysis

Analyses were carried out using Prism 6.0 software (GraphPad, La Jolla, CA, USA). Student’s *t* test was used to test differences between two samples. Analysis of variance with Dunnett’s post-test was used for multiple comparisons. *p* < 0.05 was considered statistically significant.

## Results

**Fig. 1:**
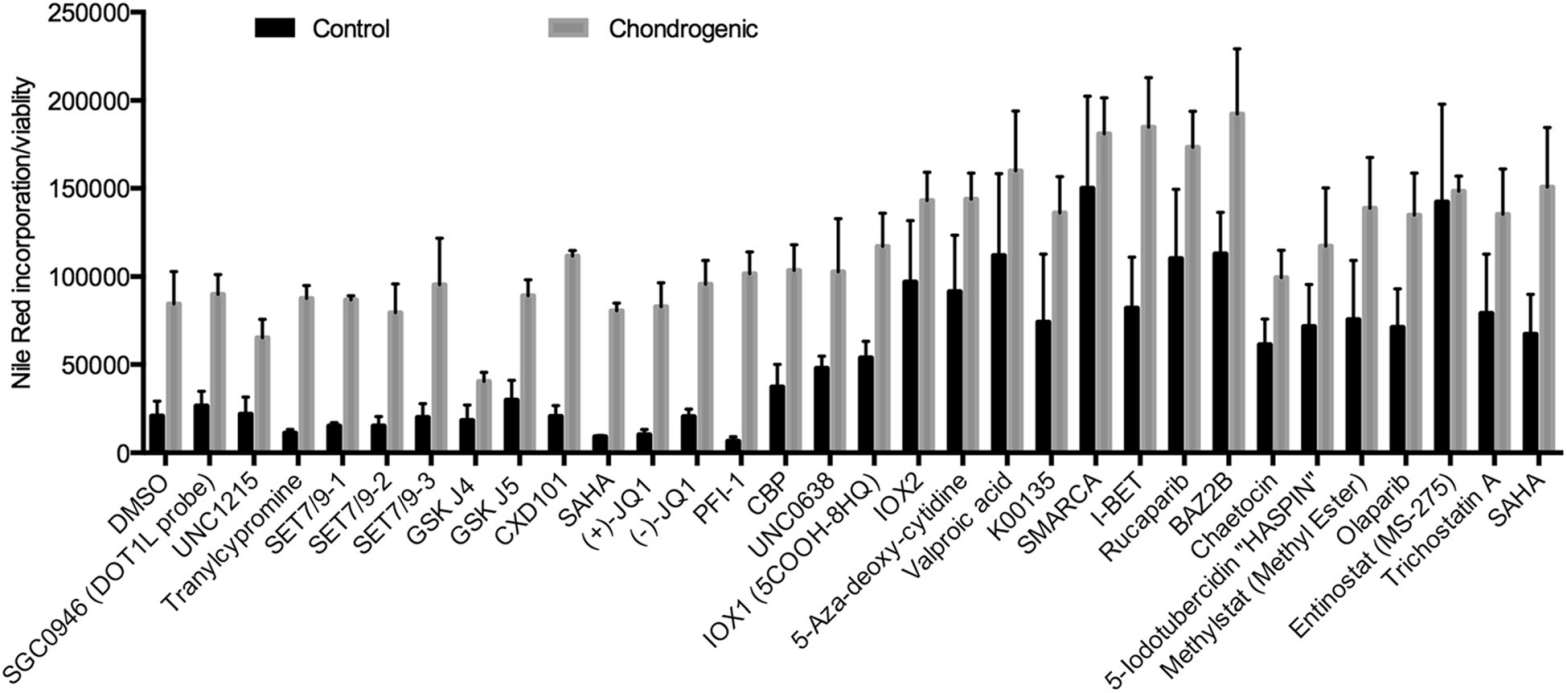
Screen of epigenetic modifying compounds in chondrogenesis. Mesenchymal stem cells were cultured with epigenetic modifying compounds for 7 days in control and chondrogenic media. Collagen output was assessed via Nile Red incorporation and normalized to viable cells using the alamarBlue assay. *DMSO* dimethyl sulphoxide

**Fig. 2:**
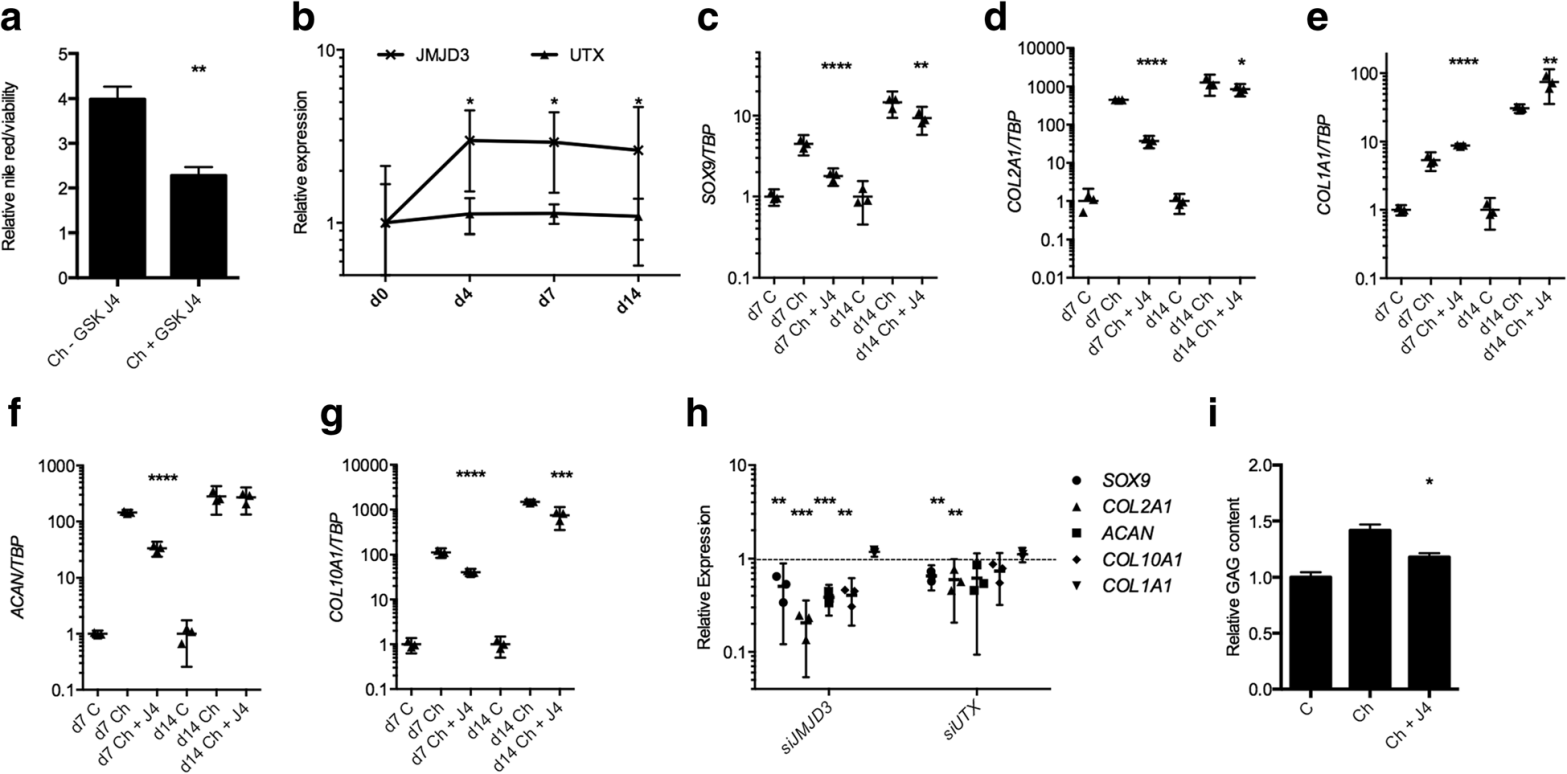
The impact of trimethylated histone 3 lysine 27 (H3K27me3) inhibition on extracellular matrix production and chondrogenic gene expression. **a** Addition of GSK-J4 to chondrogenic media of human mesenchymal stem cells (MSCs) caused a reduction in collagen production as measured by Nile Red incorporation relative to viable cells (day 7, *n* = 3 individuals, 3 technical replicates per condition). **b** Jumonji domain-containing 3 (*JMJD3*) expression was increased during chondrogenic differentiation of MSCs to form cartilage discs. Assessment of gene expression at days 7 and 14 of chondrogenesis revealed that H3K27me3 inhibition through GSK-J4 addition to chondrogenic media resulted decreased expression of SRY (sex determining region Y)-box 9 (*SOX9*), collagen type II, alpha 1 (*COL2A1*), aggrecan (*ACAN*) and collagen type X, alpha 1 (*COL10A1*) (**c**,**d**,**f**,**g**) and increased expression of collagen type I, alpha 1 (*COL1A1*) (e) (*n* = 3 individuals). **h** MSCs were pre-treated with small interfering RNA (siRNA) against *JMJD3*, lysine (K)-specific demethylase 6A (*UTX*) or non-targeting siRNA control prior to chondrogenic induction in Transwell culture. RNA was extracted and cDNA synthesized at day 7 of chondrogenesis, and expression of *SOX9*, *ACAN COL2A1*, *COL10A1* and *COL1A1* was assessed by quantitative reverse transcription polymerase chain reaction (*n* = 4 patients, *n* = 2 technical replicates per patient). *Dashed line* represents expression level following MSC treatment with non-targeting siRNA control. **i** Proteoglycan content at day 14 in cartilage discs generated in Transwell cultures was reduced following addition of GSK-J4 to chondrogenic medium (*n* = 3 individuals). *p ≤ 0.05, **p ≤ 0.01, ***p ≤ 0.001, ****p ≤ 0.0001. Data are presented as individual data points for biological replicates showing mean ± 95 % CI. **a** and **i** *t* test, **b**–**h** analysis of variance with Dunnett’s correction for multiple comparisons

Using a focused library of 31 epigenetic inhibitors in chondrogenesis of human MSCs (Fig. 1), we identified and confirmed the molecule GSK-J4 as an inhibitor of collagen output (0.63-fold; *p* = 0.0079) (Fig. 2a). No impact of GSK-J4 treatment on cell viability was detected (data not shown). GSK-J4 inhibits the KDM6 family of H3K27me3 demethylases JMJD3 and UTX. GSK-J4 has also been shown to target the KDM5 family of H3K4me3 demethylases [10].

Histone arginine or lysine residues can be mono-, di- or trimethylated. Histone 3 lysine 27 trimethylation (H3K27me3) is associated with inactive gene promoters, leading to Polycomb-mediated repression. The JMJD3 and UTX demethylases target these trimethyl groups and overcome this repression [11]. Accordingly, inhibition of UTX and JMJD3 by GSK-J4 maintains H3K27me3-driven repression of gene expression.

### JMJD3 regulates chondrogenesis

*JMJD3* but not *UTX* expression was increased at day 4 (3.0-fold, *p* = 0.017)), day 7 (2.9-fold, *p* = 0.014) and day 14 (2.6-fold, *p* = 0.0327) of chondrogenic differentiation of MSCs into cartilage discs (Fig. 2b). Consequently, the inhibition of JMJD3 and UTX activity was further explored by assessing the expression of chondrogenic markers in MSC-generated cartilage discs.

**Fig. 3:**
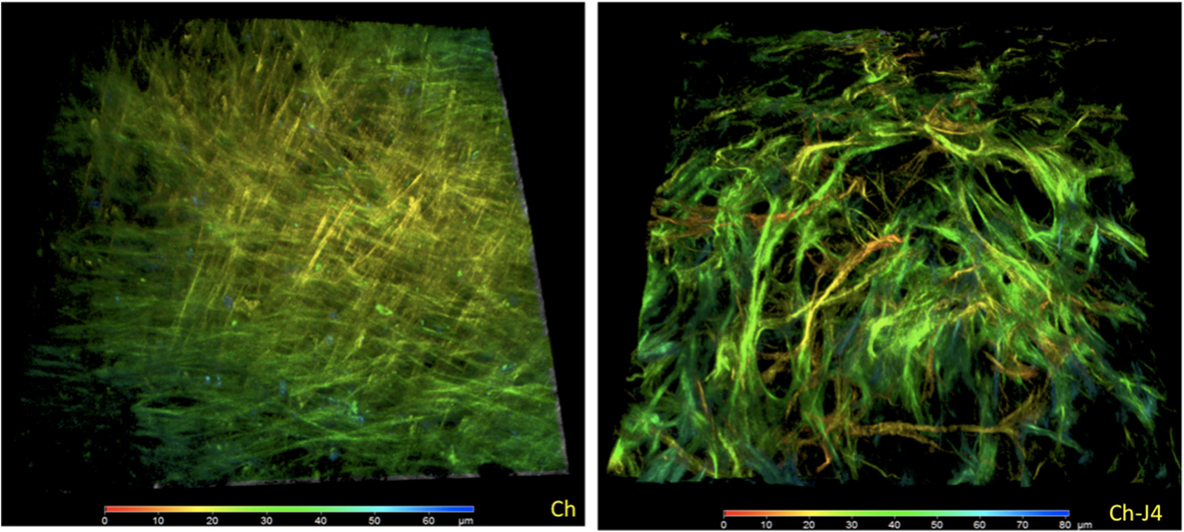
The impact of trimethylated histone 3 lysine 27 (H3K27me3) inhibition on collagen organization. Collagen organization, as assessed using second harmonic generation, was also disordered in discs generated in the presence of GSK-J4

At days 7 and 14 of chondrogenic differentiation, inhibition of H3K27me3 demethylases led to a reduction in expression of SRY (sex determining region Y)-box 9 (*SOX9*) (0.39-fold, *p* < 0.0001; 0.64-fold, *p* = 0.0019); collagen type II, alpha 1 (*COL2A1*) (0.08-fold, *p* < 0.0001; 0.67-fold, *p* = 0.0476); and collagen type X, alpha 1 (*COL10A1*) (0.36-fold, *p* < 0.0001; 0.50-fold, *p* = 0.0002). Aggrecan (*ACAN*) expression was reduced at day 7 (0.23-fold, *p* < 0.0001) and collagen type I, alpha 1 (*COL1A1*) expression was increased at days 7 and 14 (1.64-fold, *p* < 0.0001; 2.43-fold, *p* = 0.0019) (Fig. 2c–g). To test whether these effects were mediated by JMJD3 and UTX, MSCs were treated with siRNA against JMJD3 and UTX prior to Transwell induction of chondrogenesis. Expression of *ACAN*, *COL2A1*, *COL10A1* and *SOX9* was decreased, whilst expression of *COL1A1* was not altered (Fig. 2h and Additional file 3: Figure S2 show siRNA validation and treatment of MSCs with an additional siRNA against JMJD3 and UTX). Expression of adipogenic and osteogenic genes was not effected by GSK-J4 during chondrogenesis (data not shown). The glycosaminoglycan (GAG) content of cartilage discs was also reduced when GSK-J4 was present in chondrogenic medium (0.83-fold, *p* = 0.041) (Fig. 2i). SHG was used to visualize collagen fibre organization after 14 days of chondrogenic differentiation (Fig. 3). In the presence of GSK-J4, fibres were disordered and appeared thicker and more sparsely distributed.

**Fig. 4:**
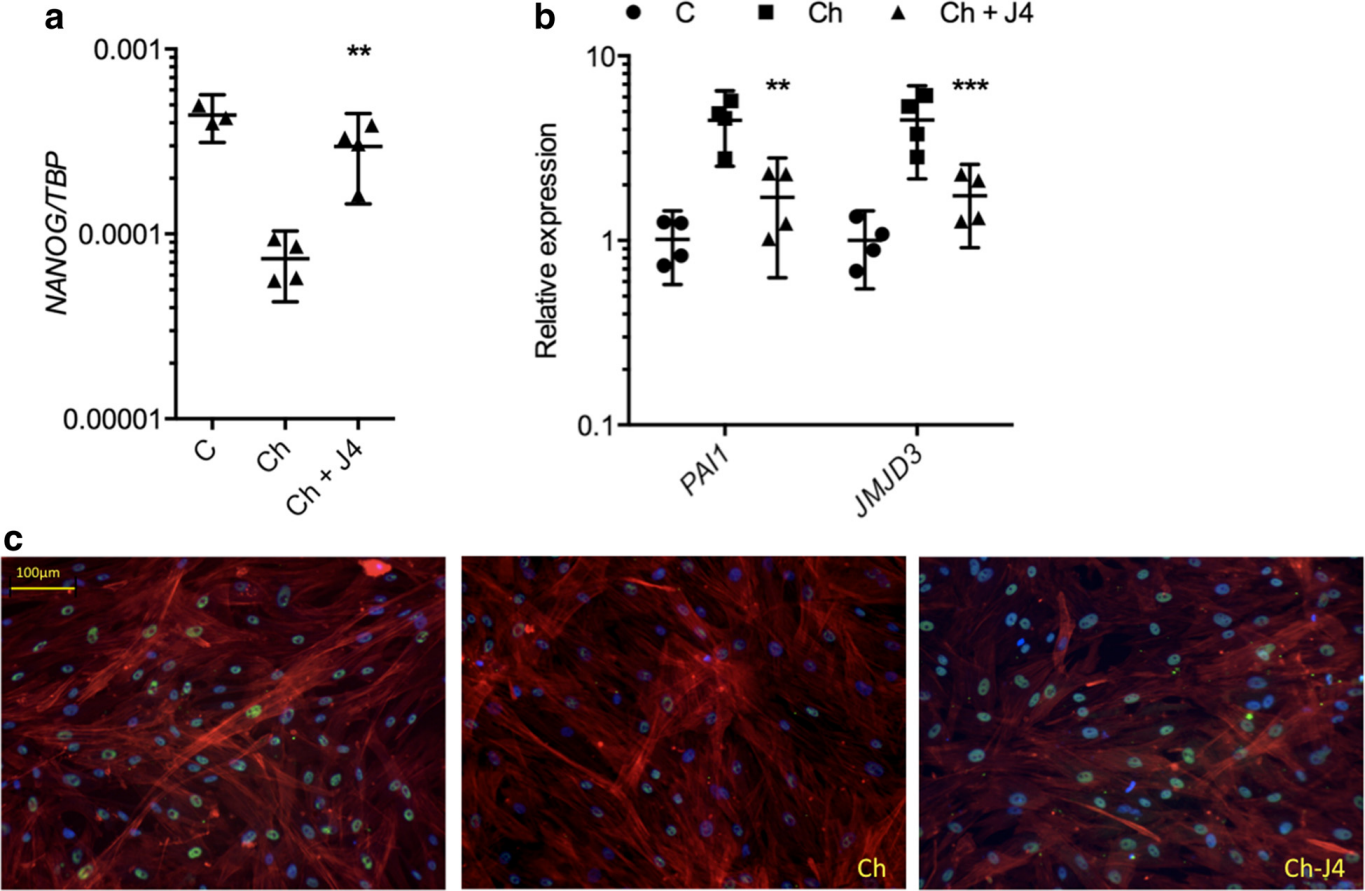
Impact of trimethylated histone 3 lysine 27 (H3K27me3) demethylase inhibition on mesenchymal stem cell (MSC) behaviour. **a** *NANOG* expression in monolayer MSCs treated for 24 h with GSK-J4 and chondrogenic medium compared with chondrogenic medium alone (*n* = 4 individuals, 4 technical replicates per condition). **b** *PAI1* and *JMJD3* expression were decreased in monolayer MSCs treated for 1 h with GSK-J4 and chondrogenic medium compared with chondrogenic medium alone (*n* = 4 individuals, 4 technical replicates per condition). **c** H3K27me3 staining (*green*) in MSCs cultured for 24 h in control medium, chondrogenic medium or chondrogenic medium plus GSK-J4. Cell cytoskeleton/actin (phalloidin, *red*) and nuclear (4′,6-diamidino-2-phenylindole, blue) staining. **p ≤ 0.01, ***p ≤ 0.001. Data are presented as individual data points for biological replicates showing mean ± 95 % CI. **a** and **b** Analysis of variance with Dunnett’s correction for multiple comparisons

We also assessed whether inhibition of H3K27me3 demethylases by GSK-J4 regulated initiation of chondrogenesis and reversion of MSCs to a less stem cell-like phenotype by assessing expression of the MSC-related gene *NANOG*. Induction of chondrogenesis caused a reduction in *NANOG* expression that was inhibited in the presence of GSK-J4 (4.0-fold, *p* = 0.0023) (Fig. 4a). TGF-β initiated MSC condensation and production of healthy cartilage, and expression of the TGF-β target gene *PAI1* by chondrogenic MSCs was inhibited by GSK-J4. Interestingly, *JMJD3* expression was also induced by TGF-β1 and inhibited in the presence of GSK-J4 (Fig. 4b).

To confirm that H3K27me3 levels are regulated in chondrogenesis, we stained chondrogenic MSCs for trimethylated H3K27. MSCs stimulated with chondrogenic medium (containing TGF-β to initiate and drive chondrogenesis) showed a reduction in H3K27me3 staining relative to control basal medium (Fig. 4c). Inhibition of JMJD3 and UTX by addition of GSK-J4 to chondrogenic medium inhibited this reduction in H3K27me3 staining.

**Fig. 5:**
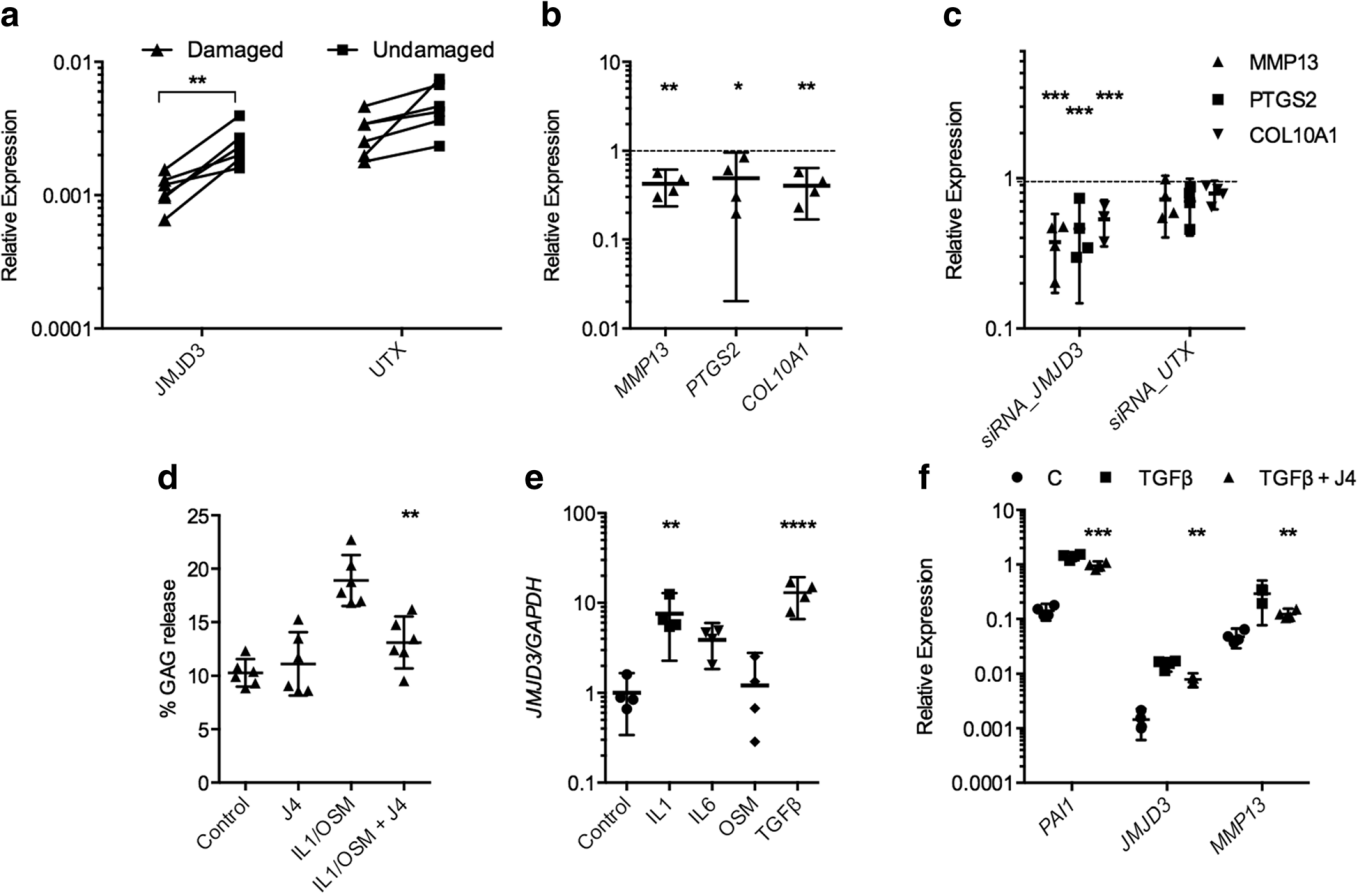
Regulation of global histone 3 lysine 27 trimethylation (H3K27me3) levels in osteoarthritis (OA) and adult articular cartilage. **a** Jumonji domain-containing 3 (*JMJD3*) was increased in damaged cartilage compared with undamaged cartilage from within the same knees of patients undergoing unicompartmental knee replacement for OA (*n* = 5 patients). **b** Inhibition of H3K27me3 demethylases by treatment of human articular chondrocyte (HACs) with GSK-J4 for 24 h caused a decrease in matrix metallopeptidase 13 (*MMP13*), prostaglandin-endoperoxide synthase 2 (*PTGS2*) and collagen type X, alpha 1 (*COL10A1*) expression (*n* = 4 patients, 4 technical replicates per condition). *Dashed line* shows control expression of each gene. **c** HACs were treated for 72 h with small interfering RNA (siRNA) against JMJD3, lysine (K)-specific demethylase 6A (UTX) and non-targeting siRNA control prior to RNA extraction and complementary DNA synthesis (*n* = 4 patients, *n* = 4 technical replicates per patient). Expression of *MMP13*, *PTGS2* and *COL10A1* was assessed by quantitative reverse transcription polymerase chain reaction. *Dashed line* shows expression level following treatment with non-targeting siRNA control. **d** Interleukin (IL)-1/oncostatin M (OSM)-induced proteoglycan loss from human articular cartilage explants was reduced in the presence of GSK-J4 (*n* = 5 patients, 3 technical replicates per condition). **e** Treatment of HACs with IL-1, IL-6 and transforming growth factor (TGF)-β increased *JMJD3* expression. **f** Expression of *PAI1*, *JMJD3* and *MMP13* following 6-h treatment of HACs with TGF-β with or without GSK-J4 (*n* = 4 patients, 4 technical replicates per condition). *p ≤ 0.05, **p ≤ 0.01, ***p ≤ 0.001, ****p ≤ 0.0001. Data are presented as individual data points for biological replicates showing mean ± 95 % CI. **a** Paired *t* test, **b**–**f** Analysis of variance with Dunnett’s correction for multiple comparisons. *GAG* glycosaminoglycan

OA is postulated to result from reversion of chondrocytes to a more developmental phenotype [12]; thus, we assessed whether there was a role for JMJD3 and UTX in OA. Expression of *JMJD3* (2.16-fold, *p* = 0.007) (Fig. 5a) but not *UTX* was increased in damaged cartilage compared with undamaged cartilage from the tibial plateau of the OA knee. GSK-J4 treatment caused a decrease in gene expression of OA-associated matrix metallopeptidase 13 (*MMP13*) (0.43-fold, *p* = 0.0032), prostaglandin-endoperoxide synthase 2 (*PTGS2*) (0.49-fold, *p* = 0.0480) and *COL10A1* (0.40-fold, *p* = 0.0023) (Fig. 5b). Expression of *MMP13*, *PTGS2* and *COL10A1* was decreased following treatment of HACs with siRNA against *JMJD3*, but there was no significant decrease in expression of these genes following knockdown of *UTX*. (Fig. 5c and Additional file 4: Figure S4 show siRNA validation and gene expression in HACs following treatment with an additional siRNA against JMJD3 and UTX.) GSK-J4 inhibited IL-1β/OSM-induced GAG release from human articular cartilage explants (0.73-fold, *p* = 0.0015) (Fig. 5d), while there was no effect of GSK-J4 alone.

To explore the factors driving *JMJD3* upregulation in damaged OA cartilage, we treated HACs with OA-relevant cytokines. The inflammatory cytokines IL-1β/OSM, which also induce MMP-13 expression, caused a significant increase in *JMJD3* expression (7.56-fold, *p* = 0.0085), as did TGF-β1 (12.97-fold, *p* < 0.0001) (Fig. 5e).

**Fig. 6:**
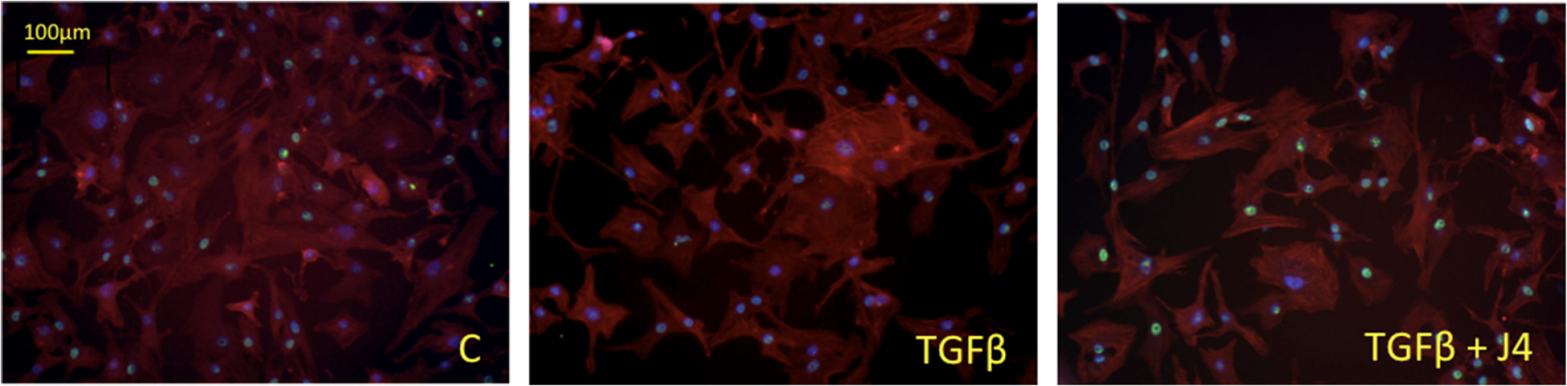
The impact of transforming growth factor (TGF)-β treatment on histone 3 lysine 27 trimethylation (H3K27me3) levels in human articular chondrocytes (HACs). H3K27me3 staining (*green*) in HACs following 1-h treatment with TGF-β or TGF-β plus GSK-J4. Cell cytoskeleton/actin (phalloidin, *red*) and nuclear (4′,6-diamidino-2-phenylindole, *blue*) staining

Given the upregulation of *JMJD3* by TGF-β in HACs and the inhibition of TGF-β-driven chondrogenesis and gene expression in MSCs, we postulated that H3K27me3 status may impact the expression of TGF-β target genes in HACs. In the presence of GSK-J4, there was a significant decrease in TGF-β-induced expression of *PAI1I* (0.68-fold, *p* = 0.0006), *JMJD3* (0.52-fold, *p* = 0.0011) and *MMP13* (0.42-fold, *p* = 0.0023) (Fig. 5f). Treatment of HACs with TGF-β reduced H3K27me3 staining, and this TGF-β-induced reduction was inhibited in the presence of GSK-J4 (Fig. 6).

## Discussion

In this study, we identified the H3K27me3 demethylases as key mediators of both chondrogenesis and OA pathogenesis. We show that inhibition of H3K27me3 demethylase activity by GSK-J4 disrupts in vitro chondrogenic differentiation of human MSCs but inhibits adult human articular cartilage degradation. GSK-J4 inhibited TGF-β-induced gene expression in both MSCs and HACs, implicating H3K27me3 demethylases in TGF-β-led regulation of chondrogenesis and OA. H3K27me3 manipulation may thus improve outcomes in cartilage tissue repair and might be a possible target for OA therapeutics.

Histone arginine or lysine residues can be mono-, di- or trimethylated. H3K27me3 is associated with inactive gene promoters, leading to Polycomb-mediated repression. The JMJD3 and UTX demethylases specifically target these trimethyl groups and overcome this repression of key transcription factors during differentiation [11]. Accordingly, GSK-J4 inhibits UTX and JMJD3 activity and thus maintains H3K27me3-driven repression of gene expression.

We show that inhibition of JMJD3 and UTX by GSK-J4 results in decreased total collagen and GAG output during chondrogenesis, as well as a reduction in expression of cartilage-associated genes, including *SOX9*. Interestingly, *COL1A1* expression was increased following GSK-J4 treatment, but not following targeted knockdown of JMJD3 and UTX by siRNA. GSK-J4 mediated inhibition of the KDM5 family of demethylases that target the H3K4me3 activating mark may explain the upregulation of *COL1A1* following GSK-J4 treatment but not following specific targeting of JMJD3 and UTX. H3K4me3 and H3K27me3 sites have been identified in collagen genes in other systems [8, 13]. The upregulation of *COL1A1* gene expression following GSK-J4 treatment, in combination with repressed *COL2A1* and *COL10A1*, may reflect the sparser, thicker and more disorganized collagen fibres visualized using SHG. Enhancing H3K27me3 demethylase activity may therefore improve in vitro cartilage tissue generation; this warrants further investigation.

The regulation of *JMJD3* but not *UTX* expression during chondrogenesis suggests that JMJD3 plays the major role, and individually targeting each enzyme using siRNA showed that both regulated chondrogenic gene expression, although UTX knockdown had a lesser effect. A recent study showed that JMJD3 promoted endochondral bone formation in mice via association with Runx2, and also reported that H3K27me3 levels decreased at the *Runx2* promoter during osteogenesis [14]. In combination with our data, this suggests that JMJD3 has multiple roles in differentiation dependent upon the context of its expression. A direct interaction of JMJD3 with *SOX9*, the key transcription factor in regulating chondrogenesis, has not been reported. However, JMJD3 potentially has both direct and indirect effects on SOX9 expression. First, H3K27me3 is present within the *SOX9* promoter in the limb bud mesenchyme and in OA cartilage [1, 15], and *SOX9*, amongst other chondrogenic marker genes, including collagens, has previously been shown to contain H3K27m e3 sites that undergo demethylation during chondrogenesis of human MSCs [16]. Therefore, demethylase activity can directly control *SOX9* expression. Indirectly, demethylation of H3K27me3 at promoter sites of accessory factors such as the *N2RF1* gene can also lead to increased *SOX9* expression via N2RF1 binding to the *SOX9* promoter [17].

To our knowledge, our present study is the first to show H3K27me3 regulation in OA, and the results suggest that upregulation of H3K27me3 demethylase activity may promote OA progression. Inhibition of H3K27me3 demethylases in adult HACs reduced expression of OA- and hypertrophy-associated *MMP13*, *COL10A1* and *PTGS2*. GAG loss from IL-1/OSM-stimulated human cartilage explants was reduced when H3K27me3 demethylase activity was inhibited. Thus, in adult cartilage, increased JMJD3 may enhance chondrocyte hypertrophy and matrix destruction, leading to OA progression. Taken together, this work suggests a protective effect of the H3K27me3 repressive mark on cartilage. The upregulation of *JMJD3* in damaged OA cartilage and by IL-1β/OSM may help drive cartilage destruction partly through upregulating expression of matrix-degrading *MMP13*.

TGF-β-induced gene expression was inhibited in both chondrogenic MSCs and adult HACs following GSK-J4 treatment. TGF-β is a pleiotropic cytokine that is essential for chondrogenesis. The inhibition of chondrogenesis in the presence of GSK-J4 may thus be mediated through its inhibition of TGF-β signalling. In OA pathogenesis, alternate TGF-β signalling pathways are activated and a dysregulated TGF-β response occurs as pathology persists [7, 18]. This dysregulation may be partially driven by direct and indirect effects of JMJD3, which is upregulated in damaged OA cartilage, on H3K27me3 status of TGF-β target genes. We also show that TGF-β increased expression of *JMJD3* itself in both chondrogenic MSCs and HACs, an observation also reported during epithelial-mesenchymal transition [19]. The H3K27me3 status and exact mechanism by which TGF-β signalling is regulated by H3K27me3 demethylases in chondrogenic MSCs and HACs require detailed mechanistic exploration both in vitro and in murine models.

GSK-J4 can target H3K4me3 demethylases of the KDM5 family as well as UTX and JMJD3. A limitation of this study is the impact of these enzymes and sites on chondrogenesis, and we did not assess chondrocyte activity. Targeted inhibition of UTX and JMJD3 by siRNA does, however, support the important role of H3K27me3 demethylases for in vitro cartilage generation and in expression of OA-associated genes, including *MMP13*, by HACs. Future work should characterize H3K4me3 methylation status alongside H3K27me3 in chondrogenesis and in OA and should target the KDM6 and KDM5 demethylases individually.

## Conclusions

In the present study, we have shown that GSK-J4 treatment and subsequent maintenance of the H3K27me3 repressive mark decreased total collagen and GAG output during chondrogenesis and reduced expression of cartilage-associated genes. Enhancing H3K27me3 demethylase activity, and thus reducing the H3K27me3 repressive mark, may therefore improve in vitro cartilage tissue generation; this warrants further investigation. In contrast, *JMJD3* was upregulated in damaged OA cartilage and H3K27me3 inhibition prevented ex vivo cartilage degradation and expression of OA-related genes.

Taken together, we provide evidence that correct regulation of H3K27me3 status by demethylases in chondrogenic precursors and chondrocytes is crucial for both chondrogenesis and maintenance of adult articular cartilage. Enhancing H3K27me3 activity, especially that of JMJD3, may improve tissue-engineered cartilage and temporally, and anatomically targeted inhibition could prevent cartilage damage in OA.

## Abbreviations

ACAN, aggrecan; cDNA, complementary DNA; COL1A1, collagen type I, alpha 1; COL2A1, collagen type II, alpha 1; COL10A1, collagen type X, alpha 1; C_t_, cycle threshold; DMSO, dimethyl sulphoxide; GAG, glycosaminoglycan; HAC, human articular chondrocyte; H3K27me3, histone 3 lysine 27 trimethylation; IL, interleukin; JMJD3, Jumonji domain-containing 3; MMLV, Moloney murine leukaemia virus; MMP-13, matrix metallopeptidase 13; MSC, mesenchymal stem cell; OA, osteoarthritis; OSM, oncostatin M; PTGS2, prostaglandin-endoperoxide synthase 2; RT-qPCR, quantitative reverse transcription polymerase chain reaction; SHG, second harmonic generation; siRNA, small interfering RNA; SOX9, SRY (sex determining region Y)-box 9; TGF-β, transforming growth factor beta; UTX, lysine (K)-specific demethylase 6A

## Additional files

Additional file 1: Figure S1. The results of treatment with GSK-5, the less active enantiomer of GSK-4, on MSCs undergoing chondrogenesis. (A-E) Assessment of gene expression at days 7 and 14 of MSC chondrogenesis to articular cartilage discs revealed following GSK-J4 and GSK-J5 treatment. (F) *NANOG* expression in monolayer MSCs treated for 24 h with GSK-J4 and GSK-J5. (G) *PAI1* and *JMJD3* expression were decreased in monolayer MSCs treated for 1 h with GSK-J4 and GSK-J5. (H) H3K27Me3 staining (green) in MSCs cultured for 24 h in control, chondrongenic or chondrogenic medium plus GSK-J4 or GSK-J5. Cell cytoskeleton/actin (phalloidin, red), nuclear staining (DAPI, blue). (TIFF 2818 kb) Additional file 2: Figure S3. The results of treatment with GSK-5, the less active enantiomer of GSK-4, on HACs. A Treatment of HAC with GSK-J5 for 24 h. B IL1/OSM-induced proteoglycan loss from human articular cartilage explants in the presence of GSK-J4 and GSK-J5. C Expression of PAI1, JMJD3 and MMP13 following 6 h treatment of HAC with TGFβ +/- GSK-J4 and GSK-J5. D H3K27me3 staining (green) in HAC following 1 h treatment with TGFβ +/- GSK-J4 or GSK-J5. Cell cytoskeleton/actin (phalloidin, red), nuclear staining (DAPI, blue). (TIFF 1712 kb) Additional file 3: Figure S2. siRNA validation qPCR on MSCs and validation chondrogenic gene expression following treatment with an additional siRNA against JMJD3 and UTX. MSC were treated with non-targeting siRNA control and two siRNA against JMJD3 (A) and UTX (B) at 2.5, 5 and 10 nM for 48 h. MTC = mock transfection control (no siRNA), NTC = no transfection control. (C) Targeting JMJD3 and UTX with additional siRNA. MSCs were pre-treated with siRNA_2 against JMJD3, UTX or non-targeting siRNA control prior to chondrogenic induction in transwell culture. RNA was extracted and cDNA synthesized at day 7 of chondrogenesis and expression of *SOX9*, *ACAN COL2A1*, *COL10A1* and *COL1A1* assessed by RT-qPCR (*n *= 4 patients, *n* =2 technical replicates per patient). Dashed line represents expression level following MSC treatment with non-targeting siRNA control. (TIFF 372 kb) Additional file 4: Figure S4. siRNA validation qPCR on HAC and validation HAC gene expression following treatment with an additional siRNA against JMJD3 and UTX. HAC were treated with non-targeting siRNA control and two siRNA against JMJD3 (A) and UTX (B) at 2.5, 5 and 10 nM for 48 h. MTC = mock transfection control (no siRNA), NTC = no transfection control. A MSCs were pre-treated with siRNA against JMJD3, UTX or scrambled non-targeting control prior to chondrogenic induction in transwell culture. RNA was extracted and cDNA synthesized at day 7 of chondrogenesis and expression of *SOX9*, *ACAN COL2A1*, *COL10A1* and *COL1A1* assessed by RT-qPCR (*n* = 4 patients, *n* = 2 technical replicates per patient). B. HAC were treated for 72 h with siRNA_2 against JMJD3, UTX and scrambled non-targeting siRNA control prior to RNA extraction and cDNA synthesis (*n* = 4 patients, *n* = 4 technical replicates per patient). Expression of *MMP13*,* PTGS2* and *COL10A1* were assessed by RT-qPCR. Dashed line shows expression level following HAC treatment with non-targeting siRNA control. *p**≤0.05, **≤0.01, ***≤0.001, ****≤0.0001. (TIFF 337 kb)

## References

[1] Kim KI, Park YS, Im GI (2013). Changes in the epigenetic status of the SOX-9 promoter in human osteoarthritic cartilage. J Bone Miner Res.

[2] Hata K, Takashima R, Amano K, Ono K, Nakanishi M, Yoshida M, Wakabayashi M, Matsuda A, Maeda Y, Suzuki Y (2013). Arid5b facilitates chondrogenesis by recruiting the histone demethylase Phf2 to Sox9-regulated genes. Nat Commun.

[3] Dawson MA, Kouzarides T (2012). Cancer epigenetics: from mechanism to therapy. Cell.

[4] Leonard CM, Fuld HM, Frenz DA, Downie SA, Massagué J, Newman SA (1991). Role of transforming growth factor-β in chondrogenic pattern formation in the embryonic limb: stimulation of mesenchymal condensation and fibronectin gene expression by exogenous TGF-β and evidence for endogenous TGF-β-like activity. Dev Biol.

[5] Yang X, Chen L, Xu X, Li C, Huang C, Deng CX (2001). TGF-β/Smad3 signals repress chondrocyte hypertrophic differentiation and are required for maintaining articular cartilage. J Cell Biol.

[6] Shen J, Li J, Wang B, Jin H, Wang M, Zhang Y, Yang Y, Im HJ, O’Keefe R, Chen D (2013). Deletion of the transforming growth factor β receptor type II gene in articular chondrocytes leads to a progressive osteoarthritis-like phenotype in mice. Arthritis Rheum.

[7] Blaney Davidson EN, Remst DF, Vitters EL, van Beuningen HM, Blom AB, Goumans MJ, van den Berg WB, van der Kraan PM (2009). Increase in ALK1/ALK5 ratio as a cause for elevated MMP-13 expression in osteoarthritis in humans and mice. J Immunol.

[8] Sun G, Reddy MA, Yuan H, Lanting L, Kato M, Natarajan R (2010). Epigenetic histone methylation modulates fibrotic gene expression. J Am Soc Nephrol.

[9] Johnson K, Zhu S, Tremblay MS, Payette JN, Wang J, Bouchez LC, Meeusen S, Althage A, Cho CY, Wu X, et al. A stem cell-based approach to cartilage repair. Science. 2012;336(6082):717–21. 10.1126/science.1215157 22491093

[10] Heinemann B, Nielsen JM, Hudlebusch HR, Lees MJ, Larsen DV, Boesen T, Labelle M, Gerlach LO, Birk P, Helin K. Inhibition of demethylases by GSK-J1/J4. Nature. 2014;514(7520):E1–2. 10.1038/nature13688 25279926

[11] Hubner MR, Spector DL (2010). Role of H3K27 demethylases Jmjd3 and UTX in transcriptional regulation. Cold Spring Harb Symp Quant Biol..

[12] Saito T, Fukai A, Mabuchi A, Ikeda T, Yano F, Ohba S, Nishida N, Akune T, Yoshimura N, Nakagawa T, et al. Transcriptional regulation of endochondral ossification by HIF-2α during skeletal growth and osteoarthritis development. Nat Med. 2010;16(6):678–86. 10.1038/nm.2146 20495570

[13] Chernov AV, Baranovskaya S, Golubkov VS, Wakeman DR, Snyder EY, Williams R, Strongin AY. Microarray-based transcriptional and epigenetic profiling of matrix metalloproteinases, collagens, and related genes in cancer. J Biol Chem. 2010;285(25):19647–59. 10.1074/jbc.M109.088153 PMC2885243 20404328

[14] Zhang F, Xu L, Xu L, Xu Q, Li D, Yang Y, Karsenty G, Chen CD. JMJD3 promotes chondrocyte proliferation and hypertrophy during endochondral bone formation in mice. J Mol Cell Biol. 2015;7(1):23–34. 10.1093/jmcb/mjv003 PMC4342687 25587042

[15] Kumar D, Lassar AB (2014). Fibroblast growth factor maintains chondrogenic potential of limb bud mesenchymal cells by modulating DNMT3A recruitment. Cell Rep.

[16] Herlofsen SR, Bryne JC, Hoiby T, Wang L, Issner R, Zhang X, Coyne MJ, Boyle P, Gu H, Meza-Zepeda LA, et al. Genome-wide map of quantified epigenetic changes during in vitro chondrogenic differentiation of primary human mesenchymal stem cells. BMC Genomics. 2013;14:105. 10.1186/1471-2164-14-105 PMC3620534 23414147

[17] Sosa MS, Parikh F, Maia AG, Estrada Y, Bosch A, Bragado P, Ekpin E, George A, Zheng Y, Lam HM, et al. NR2F1 controls tumour cell dormancy via SOX9- and RARβ-driven quiescence programmes. Nat Commun. 2015;6:6170. 10.1038/ncomms7170 PMC4313575 25636082

[18] van der Kraan PM (2014). Age-related alterations in TGF β signaling as a causal factor of cartilage degeneration in osteoarthritis. Biomed Mater Eng.

[19] Ramadoss S, Chen X, Wang CY (2012). Histone demethylase KDM6B promotes epithelial-mesenchymal transition. J Biol Chem.

