# Supplementary figures and images for: H3K27me3 demethylases regulate in vitro chondrogenesis and chondrocyte activity in osteoarthritis
Source: Arthritis Res Ther. 2016 Jul 7;18:158. doi: 10.1186/s13075-016-1053-7 (PMC4936015; doi:10.1186/s13075-016-1053-7)

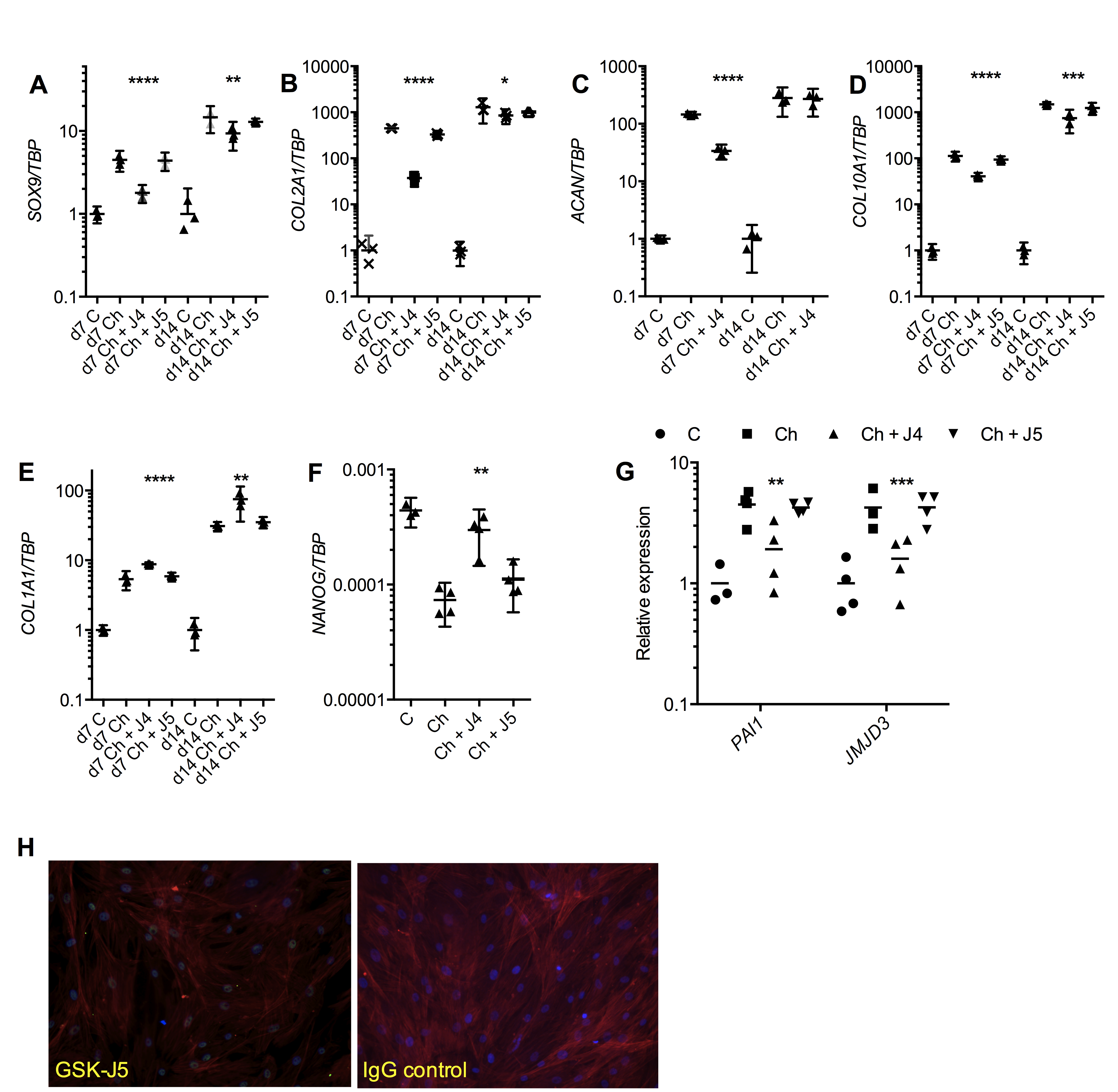

Supplement: Additional file 1: Figure S1. — The results of treatment with GSK-5, the less active enantiomer of GSK-4, on MSCs undergoing chondrogenesis. (A-E) Assessment of gene expression at days 7 and 14 of MSC chondrogenesis to articular cartilage discs revealed following GSK-J4 and GSK-J5 treatment. (F) NANOG expression in monolayer MSCs treated for 24 h with GSK-J4 and GSK-J5. (G) PAI1 and JMJD3 expression were decreased in monolayer MSCs treated for 1 h with GSK-J4 and GSK-J5. (H) H3K27Me3 staining (green) in MSCs cultured for 24 h in control, chondrongenic or chondrogenic medium plus GSK-J4 or GSK-J5. Cell cytoskeleton/actin (phalloidin, red), nuclear staining (DAPI, blue). (TIFF 2818 kb) [file 13075_2016_1053_MOESM1_ESM.tiff]

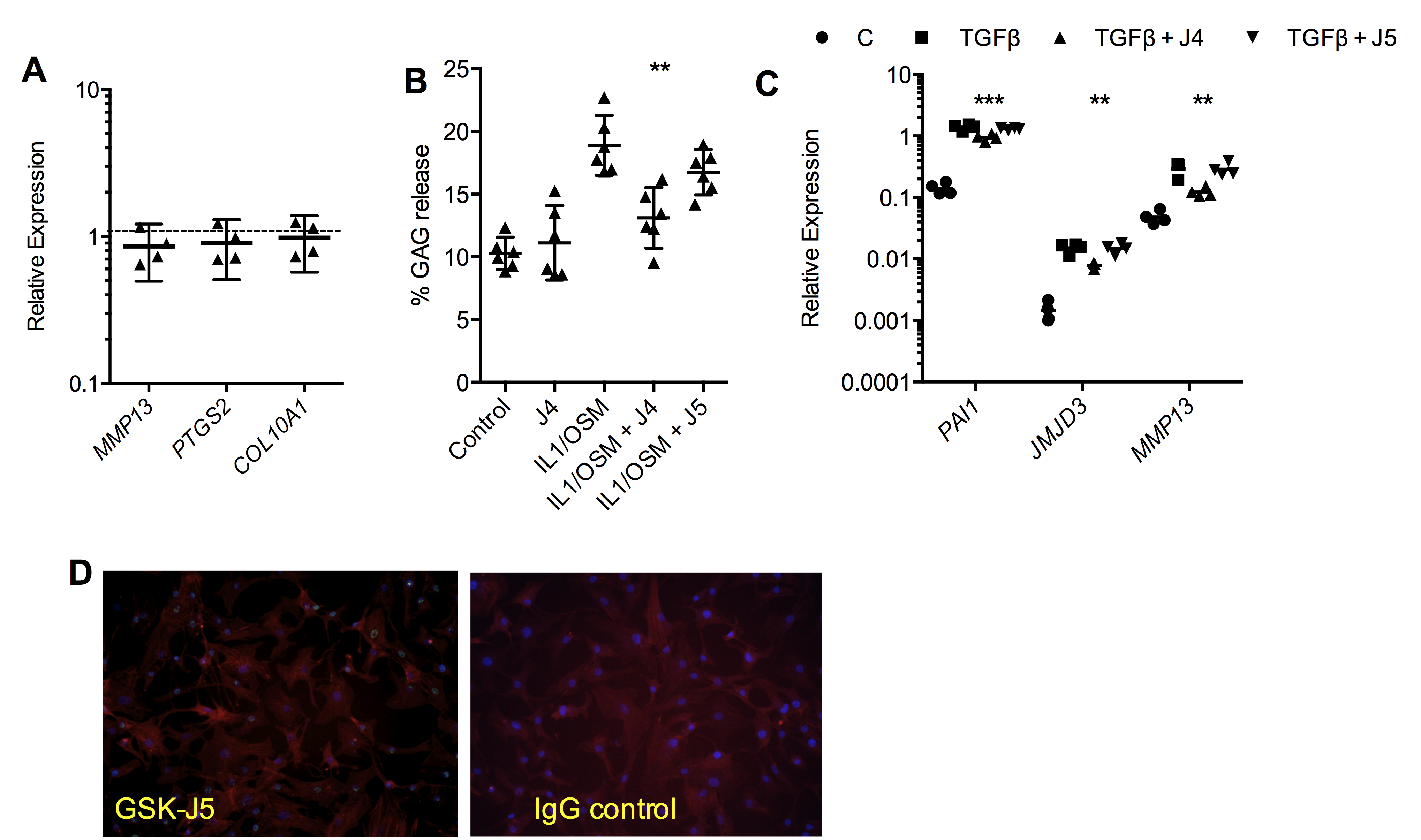

Supplement: Additional file 2: Figure S3. — The results of treatment with GSK-5, the less active enantiomer of GSK-4, on HACs. A Treatment of HAC with GSK-J5 for 24 h. B IL1/OSM-induced proteoglycan loss from human articular cartilage explants in the presence of GSK-J4 and GSK-J5. C Expression of PAI1, JMJD3 and MMP13 following 6 h treatment of HAC with TGFβ +/- GSK-J4 and GSK-J5. D H3K27me3 staining (green) in HAC following 1 h treatment with TGFβ +/- GSK-J4 or GSK-J5. Cell cytoskeleton/actin (phalloidin, red), nuclear staining (DAPI, blue). (TIFF 1712 kb) [file 13075_2016_1053_MOESM2_ESM.tiff]

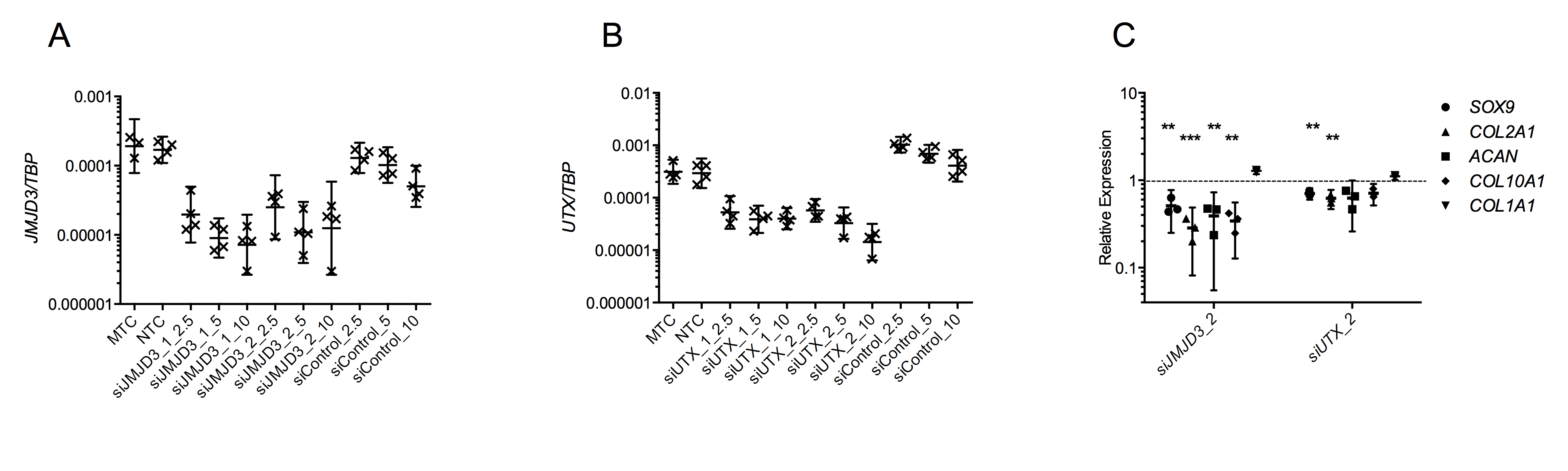

Supplement: Additional file 3: Figure S2. — siRNA validation qPCR on MSCs and validation chondrogenic gene expression following treatment with an additional siRNA against JMJD3 and UTX. MSC were treated with non-targeting siRNA control and two siRNA against JMJD3 (A) and UTX (B) at 2.5, 5 and 10 nM for 48 h. MTC = mock transfection control (no siRNA), NTC = no transfection control. (C) Targeting JMJD3 and UTX with additional siRNA. MSCs were pre-treated with siRNA_2 against JMJD3, UTX or non-targeting siRNA control prior to chondrogenic induction in transwell culture. RNA was extracted and cDNA synthesized at day 7 of chondrogenesis and expression of SOX9, ACAN COL2A1, COL10A1 and COL1A1 assessed by RT-qPCR (n = 4 patients, n =2 technical replicates per patient). Dashed line represents expression level following MSC treatment with non-targeting siRNA control. (TIFF 372 kb) [file 13075_2016_1053_MOESM3_ESM.tiff]

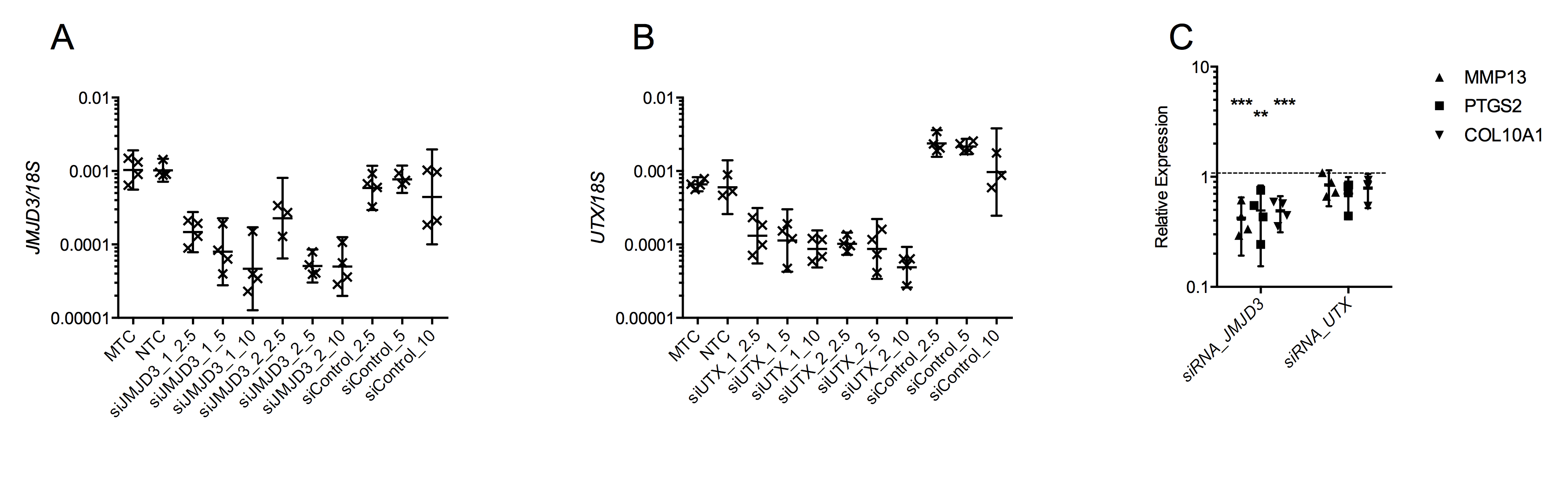

Supplement: Additional file 4: Figure S4. — siRNA validation qPCR on HAC and validation HAC gene expression following treatment with an additional siRNA against JMJD3 and UTX. HAC were treated with non-targeting siRNA control and two siRNA against JMJD3 (A) and UTX (B) at 2.5, 5 and 10 nM for 48 h. MTC = mock transfection control (no siRNA), NTC = no transfection control. A MSCs were pre-treated with siRNA against JMJD3, UTX or scrambled non-targeting control prior to chondrogenic induction in transwell culture. RNA was extracted and cDNA synthesized at day 7 of chondrogenesis and expression of SOX9, ACAN COL2A1, COL10A1 and COL1A1 assessed by RT-qPCR (n = 4 patients, n = 2 technical replicates per patient). B. HAC were treated for 72 h with siRNA_2 against JMJD3, UTX and scrambled non-targeting siRNA control prior to RNA extraction and cDNA synthesis (n = 4 patients, n = 4 technical replicates per patient). Expression of MMP13, PTGS2 and COL10A1 were assessed by RT-qPCR. Dashed line shows expression level following HAC treatment with non-targeting siRNA control. p*≤0.05, **≤0.01, ***≤0.001, ****≤0.0001. (TIFF 337 kb) [file 13075_2016_1053_MOESM4_ESM.tiff]
